# Assessing the Well-Being at Work of Nurses and Doctors in Hospitals: Protocol for a Scoping Review of Monitoring Instruments

**DOI:** 10.2196/43692

**Published:** 2023-08-25

**Authors:** Amber Boskma, Kim van der Braak, Neda Ansari, Lotty Hooft, Götz Wietasch, Arie Franx, Maarten van der Laan

**Affiliations:** 1 Department of Surgery University Medical Center Groningen Groningen Netherlands; 2 Netherlands Federation of University Medical Centres Utrecht Netherlands; 3 Cochrane Netherlands University Medical Center Utrecht Utrecht Netherlands; 4 Julius Center for Health Sciences and Primary Care University Medical Center Utrecht Utrecht Netherlands; 5 Department of Anesthesiology University Medical Center Groningen Groningen Netherlands; 6 Department of Obstetrics and Gynecology Erasmus University Medical Center Rotterdam Netherlands

**Keywords:** well-being at work, well-being, well being, health care professionals, doctors, nurses, monitoring, assessment, measure, scale, instruments, scoping literature review, occupational health

## Abstract

**Background:**

Well-being at work can be defined as “creating an environment to promote a state of contentment which allows an employee to flourish and achieve their full potential for the benefit of themselves and their organisation.” In the health care context, well-being at work of nurses and doctors is important for good patient care. Moreover, it is strongly associated with individual- and organization-level consequences. Relevant literature presents models and concepts of physical, mental, and social well-being. This study uses the 6 elements of the job demands-resources (JD-R) model to interpret well-being at work (job demands, job resources, personal resources, leadership, well-being, and outcomes) as part of a Netherlands Federation of University Medical Hospitals program to find ways to improve and monitor health care professionals’ well-being in Dutch hospitals. Many instruments exist to measure well-being at work in terms of population, setting, and other aspects. An overview of available and eligible instruments assessing and monitoring the well-being of nurses and doctors is currently missing.

**Objective:**

We will perform a scoping review aiming to provide an overview of validated instruments assessing and monitoring the well-being of nurses and doctors at work.

**Methods:**

We will perform a search of published literature in the following databases: Medline, Embase, and CINAHL. Studies will be eligible if they (1) assess well-being at work of nurses and doctors employed in hospitals; (2) describe an evaluation of an instrument or review an instrument; (3) measure well-being at work or aspects of well-being at work according to the elements of the JD-R model, and (4) were published in English from 2011 onwards. Title/abstract screening according to the eligibility criteria will be followed by full-text screening. Data extraction of included studies will be conducted by 3 reviewers independently. Reviewers will use standardized data extraction forms that include study characteristics, sample characteristics, measurement instrument details, and psychometric properties. The analysis will be descriptive. When synthesizing the data, a distinction will be made between comprehensive instruments and common instruments.

**Results:**

This scoping review identifies instruments that have been developed and validated for monitoring the well-being of nurses and doctors at work. Studies were searched between September and December 2021 and screened between December 2021 and May 2022. A total of 739 studies were included.

**Conclusions:**

Timely screening of well-being at work may be beneficial for individual health care workers, the organization, and patients. There is often a substantial gap and mismatch between employer perceptions of well-being and well-being interventions. It is important to develop and implement suitable interventions adapted to the needs of nurses and doctors and their health or other problems. Well-being screening should be timely to gain insight into these needs and problems. Moreover, to determine the effectiveness of well-being interventions, measurement is mandatory. The results will be critical for organizations to select a monitoring instrument that best fits the needs of employees and organizations.

**International Registered Report Identifier (IRRID):**

DERR1-10.2196/43692

## Introduction

Well-being at work for nurses and doctors employed in hospitals is an important condition for achieving effective, safe, and good patient care [1-3]. Moreover, well-being at work is strongly associated with serious consequences at an individual level, such as poor work-life balance [3,4], obesity [3], reduced quality of life of the health care worker [3,5], substance abuse, and suicide [3,6]. At an organizational level it is related to high staff turnover [3,6], absenteeism [3,7], and high health care costs [3].

The Chartered Institute of Personnel and Development defines well-being at work as “creating an environment to promote a state of contentment which allows an employee to flourish and achieve their full potential for the benefit of themselves and their organisation” [8]. Well-being comprises psychological well-being, physical well-being, and social well-being [9]. Also, a variety of published literature presents models and concepts of well-being. Deci et al [10] reported the self-determination theory. The self-determination theory suggests that both employees’ performance and their well-being are affected by the type of motivation they have experienced [10]. For medical students, the “coping reserve tank” was illustrated [11]. This is a coping reservoir that can be replenished or drained by various factors, such as stress, mentorship, time demands, and support [11]. Potential outcomes were described as resilience versus burnout [11]. Schaufeli [12] developed the job demands-resources model (JD-R model) facilitating communication about “work and well-being.” In essence, the JD-R model integrates 2 processes: the stress process, which is sparked by excessive job demands and lack of resources, and a motivational process, which is triggered by abundant job resources and may lead to positive outcomes, such as organizational commitment, intention to stay, and work performance [12]. Thus, different components, including resources (eg, support, development opportunities, team atmosphere), demands (eg, stress, workload, conflicts), and personal resources (eg, leadership, intrinsic motivation, resilience), contribute to positive well-being (eg, job satisfaction) or negative well-being (eg, burnout). Abundant resources and reasonable demands will result in positive outcomes (eg, performance, commitment); the reverse will lead to negative outcomes (sickness, absenteeism) [12].

Measuring well-being at work among doctors and nurses is not easy, since this multidimensional concept encompasses diverse elements [8,13]. Instruments to measure well-being at work vary in specific professions [14] and specific settings [15] or include only one or two aspects of well-being at work [12,16]. This study is part of a program of the Netherlands Federation of University Medical Hospitals (NFU) about finding ways to improve and monitor health care professionals’ well-being in Dutch hospitals. Strikingly, there is often a substantial gap and mismatch between employer perceptions of well-being and well-being interventions [8]. It is important to develop and implement suitable interventions adapted to the needs of nurses and doctors and their health and other problems [8,17]. Well-being screening should be timely to gain insight into these needs and problems. Moreover, measurement to determine the effectiveness of well-being interventions is mandatory [8,17].

To summarize, when hospitals assess and monitor the well-being of nurses and doctors with validated instruments, they can, in a timely manner, design and start tailored interventions to prevent negative well-being in the workplace, thereby contributing to sustainable employability and quality of care [18,19]. An overview of available and eligible instruments assessing and monitoring the well-being of nurses and doctors is currently missing. Scoping reviews are helpful to explore broad, complex, and heterogeneous literature [20]. For this study, a scoping review is planned to obtain an overview of the field of well-being instruments and identify its breadth.

## Methods

### Aim

This scoping review aims to identify instruments monitoring the well-being at work of nurses and doctors working in hospitals. The Preferred Reporting Items for Systematic reviews and Meta-Analyses extension for Scoping Reviews (PRISMA-ScR) [21] and the 6-stage framework of Arksey and O’Malley [22] for conducting scoping reviews will be used.

### Information Sources

A priori, possible previous reviews of this topic were checked. Published articles will be searched in the following electronic databases: Medline, Embase and CINAHL. To avoid including outdated literature, we will include only articles published after 2011 [23]. To identify instruments that are applied in present clinical practice, preference is given to more-frequent, lower-volume searches to fit the exponentially changing field of health care [24,25]. Gray literature will not be used as an information source.

### Search Strategy

The search strategy was created by AB with assistance by an information specialist and the research team. Several terms derived from the research aim were identified to develop search strings to find relevant literature. Keywords and Medical Subject Heading (MeSH) terms related to the domain (nurses and doctors working in hospitals), the determinants (instruments for monitoring), and the outcome (well-being at work) will be used. The first draft of the search string was based on 5 relevant, previously published “golden bullets,” sample articles that had to be found in the data set. Afterwards, a test data set was screened by the first author to optimize the search string. After this screening, the data set turned out to be too large (more than 17,000 studies), so to ensure the feasibility of the study it was decided to make the search more specific. For the search strategy see Tables 1, 2, and 3.

**Table 1:** Search strategy for Medline.

ID	Query	Results
1	(“Health care professionals” or Caregivers or “Health care providers” or Practitioners or Doctor or nurse? or physician? or resident? or “health care worker” or “health staff”).ti. or exp *”physicians”/ or exp *“Medical staff”/ or *“Residents”/ or exp *“Nurses”/ or exp *“Nursing Staff”/	461,194
2	(instrumentation or methods).fs. or exp “psychometrics”/ or psychometr*.ti,ab. or clinimetr*.tw. or clinometr*.tw. or exp “Health Status Indicators”/ or survey?.ti,ab. or score.ti,ab. or scale.ti,ab. or subscale.ti,ab. or (measurement adj 3 instrument).ti,ab. or subscale*.ti,ab. or item-discriminant.ti,ab. or interscale correlation*.ti,ab. or “ceiling effect.”ti,ab. or “floor effect.”ti,ab. or “Item response model.”ti,ab. or Rasch.ti,ab. or “Differential item functioning.”ti,ab. or “item bank.”ti,ab. or (item adj3 (correlation* or selection* or reduction* or bank)).ti,ab.	6,142,766
3	Validation Studies.pt. or exp “observer variation”/ or observer variation.ti,ab. or exp “reproducibility of results”/ or exp “discriminant analysis”/ or valid*.ti,ab. or (cronbach* adj3 (alpha or alphas)).ti,ab. or interrater.ti,ab. or inter-rater.ti,ab. or intrarater.ti,ab. or intra-rater.ti,ab. or intertester.ti,ab. or inter-tester.ti,ab. or intratester.ti,ab. or intra-tester.ti,ab. or interobserver.ti,ab. or inter-observer.ti,ab. or intraobserver.ti,ab. or intraobserver.ti,ab. or interexaminer.ti,ab. or inter-examiner.ti,ab. or intraexaminer.ti,ab. or intra-examiner.ti,ab. or interindividual.ti,ab. or inter-individual.ti,ab. or intraindividual.ti,ab. or intra-individual.ti,ab. or kappa.ti,ab. or kappa?s.ti,ab. or kappas.ti,ab. or ((replicab* or repeated) and (measure or measures or findings or result or results or test or tests)).ti,ab. or concordance.ti,ab. or (intraclass and correlation*).ti,ab. or (uncertainty and (measurement or measuring)).ti,ab. or “standard error of measurement.”ti,ab. or sensitiv*.ti,ab.	2,822,376
4	exp Burnout, Psychological/ or exp Personal Satisfaction/ or exp Mental Health/ or (satisfaction or well-being or fulfilment or burnout or ((psychological or mental) adj health) or thriving or environment or ethic*).ti,ab,kf.	1,122,990
5	1, 2, 3, and 4	3301

**Table 2:** Search strategy for Embase.

ID	Query^a^	Results
S1	S17 AND S10 AND S4 AND S3	1329
S2	S17 AND S10 AND S4 AND S3	229
S3	S9 OR S8 OR S7 OR S6 OR S5	46,447
S4	S25 OR S24 OR S23 OR S22	421,434
S5	TI(satisfaction or well-being or fulfilment or burnout or ((psychological or mental) N1 health) or thriving or environment or ethic*)	145,799
S6	AB(satisfaction or well-being or fulfilment or burnout or ((psychological or mental) N1 health) or thriving or environment or ethic*)	36,566
S7	(MH “Mental Health”)	44,106
S8	(MH “Job Satisfaction”) OR (MH “Personal Satisfaction”)	35,259
S9	(MH “Burnout, Professional”)	12,506
S10	S15 OR S14 OR S13 OR S12 OR S11	502,496
S11	TI(valid* or (cronbach* N3 (alpha or alphas)) or interrater or inter-rater or intrarater or intra-rater or intertester or inter-tester or intratester or intra-tester or interobserver or inter-observer or intraobserver or intraobserver or interexaminer or inter-examiner or intraexaminer or intra-examiner or interindividual or inter-individual or intraindividual or intra-individual or kappa or kappa?s or kappas or ((replicab* or repeated) and (measure or measures or findings or result or results or test or tests)) or concordance or (intraclass and correlation*) or (uncertainty and (measurement or measuring)) or “standard error of measurement” or sensitiv*)	80,499
S12	AB(valid* or (cronbach* N3 (alpha or alphas)) or interrater or inter-rater or intrarater or intra-rater or intertester or inter-tester or intratester or intra-tester or interobserver or inter-observer or intraobserver or intraobserver or interexaminer or inter-examiner or intraexaminer or intra-examiner or interindividual or inter-individual or intraindividual or intra-individual or kappa or kappa?s or kappas or ((replicab* or repeated) and (measure or measures or findings or result or results or test or tests)) or concordance or (intraclass and correlation*) or (uncertainty and (measurement or measuring)) or “standard error of measurement” or sensitiv*)	417,988
S13	(MH “Kappa Statistic”)	17,505
S14	(MH “Reproducibility of Results”)	67,821
S15	(MH “Interrater Reliability”)	27,493
S16	S25 OR S24 OR S23 OR S22 OR S21	82,033
S17	S20 OR S19 OR S18	753,761
S18	TI(clinimetr* or clinometr* or psychometr* or survey? or score or scale or subscale or (measurement N3 instrument) or subscale* or item-discriminant or interscale correlation* or “ceiling effect” or “floor effect” or “Item response model” or Rasch or “Differential item functioning” or “item bank” or (item N3 (correlation* or selection* or reduction* or bank)))	12,483
S19	AB(clinimetr* or clinometr* or psychometr* or survey? or score or scale or subscale or (measurement N3 instrument) or subscale* or item-discriminant or interscale correlation* or “ceiling effect” or “floor effect” or “Item response model” or Rasch or “Differential item functioning” or “item bank” or (item N3 (correlation* or selection* or reduction* or bank)))	694,621
S20	(MH “Psychometrics”) OR (MH “Measurement Issues and Assessments”)	32,107
S21	AB(“Healthcare professionals” or Caregivers or “Healthcare providers” or Practitioners or Doctor or nurse? or physician? or resident? or “healthcare worker” or “health staff”)	556,739
S22	TI(“Healthcare professionals” or Caregivers or “Healthcare providers” or Practitioners or Doctor or nurse? or physician? or resident? or “healthcare worker” or “health staff”)	296,467
S23	(MM “Nurses+”)	141,674
S24	(MM “Medical Staff, Hospital+”)	3977
S25	(MM “Physicians+”)	64,766

^a^The following abbreviations are applicable to the queries: T1: title; N1: adjacency of 1 word; AB: abstract; MH: mesh heading; MM: major mesh.

**Table 3:** Search strategy for CINAHL.

ID	Query^a^	Results
S1	S17 AND S10 AND S4 AND S3	1329
S2	S17 AND S10 AND S4 AND S3	229
S3	S9 OR S8 OR S7 OR S6 OR S5	46,447
S4	S25 OR S24 OR S23 OR S22	421,434
S5	TI(satisfaction or well-being or fulfilment or burnout or ((psychological or mental) N1 health) or thriving or environment or ethic*)	145,799
S6	AB(satisfaction or well-being or fulfilment or burnout or ((psychological or mental) N1 health) or thriving or environment or ethic*)	36,566
S7	(MH “Mental Health”)	44,106
S8	(MH “Job Satisfaction”) OR (MH “Personal Satisfaction”)	35,259
S9	(MH “Burnout, Professional”)	12,506
S10	S15 OR S14 OR S13 OR S12 OR S11	502,496
S11	TI(valid* or (cronbach* N3 (alpha or alphas)) or interrater or inter-rater or intrarater or intra-rater or intertester or inter-tester or intratester or intra-tester or interobserver or inter-observer or intraobserver or intraobserver or interexaminer or inter-examiner or intraexaminer or intra-examiner or interindividual or inter-individual or intraindividual or intra-individual or kappa or kappa?s or kappas or ((replicab* or repeated) and (measure or measures or findings or result or results or test or tests)) or concordance or (intraclass and correlation*) or (uncertainty and (measurement or measuring)) or “standard error of measurement” or sensitiv*)	80,499
S12	AB(valid* or (cronbach* N3 (alpha or alphas)) or interrater or inter-rater or intrarater or intra-rater or intertester or inter-tester or intratester or intra-tester or interobserver or inter-observer or intraobserver or intraobserver or interexaminer or inter-examiner or intraexaminer or intra-examiner or interindividual or inter-individual or intraindividual or intra-individual or kappa or kappa?s or kappas or ((replicab* or repeated) and (measure or measures or findings or result or results or test or tests)) or concordance or (intraclass and correlation*) or (uncertainty and (measurement or measuring)) or “standard error of measurement” or sensitiv*)	417,988
S13	(MH “Kappa Statistic”)	17,505
S14	(MH “Reproducibility of Results”)	67,821
S15	(MH “Interrater Reliability”)	27,493
S16	S25 OR S24 OR S23 OR S22 OR S21	82,033
S17	S20 OR S19 OR S18	753,761
S18	TI(clinimetr* or clinometr* or psychometr* or survey? or score or scale or subscale or (measurement N3 instrument) or subscale* or item-discriminant or interscale correlation* or “ceiling effect” or “floor effect” or “Item response model” or Rasch or “Differential item functioning” or “item bank” or (item N3 (correlation* or selection* or reduction* or bank)))	12,483
S19	AB(clinimetr* or clinometr* or psychometr* or survey? or score or scale or subscale or (measurement N3 instrument) or subscale* or item-discriminant or interscale correlation* or “ceiling effect” or “floor effect” or “Item response model” or Rasch or “Differential item functioning” or “item bank” or (item N3 (correlation* or selection* or reduction* or bank)))	694,621
S20	(MH “Psychometrics”) OR (MH “Measurement Issues and Assessments”)	32,107
S21	AB(“Healthcare professionals” or Caregivers or “Healthcare providers” or Practitioners or Doctor or nurse? or physician? or resident? or “healthcare worker” or “health staff”)	556,739
S22	TI(“Healthcare professionals” or Caregivers or “Healthcare providers” or Practitioners or Doctor or nurse? or physician? or resident? or “healthcare worker” or “health staff”)	296,467
S23	(MM “Nurses+”)	141,674
S24	(MM “Medical Staff, Hospital+”)	3977
S25	(MM “Physicians+”)	64,766

^a^The following abbreviations are applicable to the queries: T1: title; N1: adjacency of 1 word; AB: abstract; MH: mesh heading; MM: major mesh.

### Inclusion Criteria

Studies will be eligible if they (1) assess well-being of nurses and doctors working in hospitals; (2) describe an evaluation of an instrument or review an instrument; (3) measure well-being at work or aspects of well-being at work according the elements of the JD-R model and (4) were published in English in or after 2011.

### Exclusion Criteria

Studies will be excluded if they (1) describe only the development of the instrument but not its evaluation or (2) have a sample that consists only of students, without employees.

### Data Management

Records and data will be managed by using the software Endnote (version 20.1; Clarivate Analytics), Rayyan (Rayyan Systems), EPPI-Reviewer (EPPI-center) and Mendeley Reference Manager (version 2.590; Elsevier).

### Selection Process

Study screening and selection will be conducted independently by AB, KvdB, and NA using Rayyan. After the removal of duplicate records identified by the search strategy, articles will be screened on titles and abstracts for the inclusion and exclusion criteria. To ensure consistent screening, the first 100 articles will be pilot-screened by all 3 researchers until consensus is reached. After validation, the hits will be allocated to 1 of the 3 researchers. Eligible articles will then be assessed on their full text. To ensure consistent full-text screening, the first 18 articles will be pilot-screened by all 3 researchers until consensus is reached. After validation, the full texts will be subdivided among the 3 researchers. Justification for study inclusion and exclusion will be reported. Inclusion reasons and exclusion reasons will be marked with a label. Uncertainty in selection and the data extraction process will be resolved by consensus [12]. The JD-R model will be used to assess if the reported instruments appropriately measured well-being or aspects of well-being at work (job demands, job resources, engaged leadership, personal resources, employees’ well-being, outcomes) [12]. The energy compass of the JD-R model includes all aspects of well-being at work and is applicable to different employees in different settings [12]. Included instruments will be categorized according to the 6 domains of the JD-R model. The domain “job demands” contains the categories “qualitative job demands,” “quantitative job demands,” and “organizational demands.” The domain “job resources” contains the categories “social resources,” “work resources,” “organizational resources,” and “developmental resources.” The domain “engaged leadership” contains the categories “inspiring,” “strengthening,” and “connecting.” The domain “personal resources” contains the categories “resilience,” “self-efficacy,” “optimism,” flexibility,” “setting one’s own limits,” “productivity,” “goal direction,” and “self-development.” The domain “well-being” contains the positive and negative categories “burnout,” “work engagement,” “psychological distress,” “boredom,” “sleep problems,” and “job satisfaction.” The domain “outcomes” contains the categories “commitment,” “employability,” and “performance.” Study search and selection will be illustrated using the PRISMA flow diagram.

### Data Collection Process

Data extraction and full-text screening will be conducted at the same time. AB, KvdB, and NA will independently use the standardized data extraction form, which will be developed a priori in Excel (Microsoft Corp). The elements that will be charted for each article are (1) study characteristics, including year, authors, country, study ID, and study aim (validation study, measuring variables, both, other); (2) sample characteristics, including type of health care professionals (nurses, doctors, other), setting (hospital, partly hospital), and sample size; (3) measurement instrument, including the name of the instrument, main construct measured, subconstructs measured, job demands, job resources, leadership, personal resources, well-being, and outcomes; and (4) psychometric properties, including validity, reliability, responsiveness, and quality references. The aim of this study is not to evaluate psychometric properties but include only the evaluated instruments. Therefore, information about psychometric properties (whether validity, reliability, and responsiveness are tested) will be extracted. When no psychometric properties are reported, the references mentioned for evaluation will be extracted. Table 4 shows further details.

All instruments meeting the inclusion criteria will be extracted from studies. If more than one instrument has been described, instrument details will be extracted separately for each instrument. The data extraction form will be tested by AB, KvdB, and NA on 18 studies to ensure adequacy of the extraction. Disagreements will be discussed, and the form will be refined, if necessary, after the pilot phase with the 18 studies.

**Table 4:** Defining variables.

Item			Definition
**1. Study characteristics**			
	Year	Picklist Years from 2011, up to and including 2021	
	Authors	Last name, initials	
	Country	Country where participants are recruited	
	Study ID	Unique code to identify the study, found in Eppi-reviewer	
	Study aim	Picklist Validation study: the study tests or evaluates instruments Measuring variables: the study uses instruments to measure variables Both: studies that both validate instruments and measure variables Other: studies in which the above is not described (eg, study protocols)	
**2. Sample characteristics**			
	Health care professionals	Picklist Nurses (exclusively or mixed with other types of participants): all kinds of nurses providing direct patient care in hospitals (eg, intensive care nurses and pediatric nurses) Doctors (exclusively or mixed with other types of participants): all kinds of doctors providing direct patient care in hospitals (eg, residents, medical assistants, and medical specialists in various specialties) Both: studies in which both nurses and doctors are described	
	Setting	Picklist Hospital: full sample works in hospital Partly in hospital: a part of the sample works in a hospital; any kind of hospital will be included (eg, psychiatric hospitals, general hospitals, and academic hospitals)	
	Sample size	Full sample size: if only part of the sample works in a hospital, the full sample needs to be reported	
**3. Measurement instrument**			
	Name of instrument	Full name of the instrument, including any abbreviations (eg, Professional Quality of Life Scale, version 5; ProQol-5).	
	Main construct measured	Main construct intended to be measured (eg, professional quality of life)	
	Subconstructs measured	Subthemes intended to be measured (eg, compassion satisfaction, secondary traumatic stress, burnout)	
	JD-R^a^ demands	Picklist Job demands: the outcome fits with “job demands” according to the JD-R model. N/A^b^: the outcome does not fit with “job demands” according to the JD-R model	
	JD-R resources	Picklist Job resources: the outcome fits with “job resources” according to the JD-R model N/A: the outcome does not fit with “job resources” according to the JD-R model	
	JD-R leadership	Picklist Engaged leadership: the outcome fits with “engaged leadership” according to the JD-R model N/A: the outcome does not fit with “engaged leadership” according to the JD-R model	
	JD-R well-being	Picklist Employee well-being: the outcome fits with “employee well-being” according to the JD-R model N/A: the outcome does not fit with “employee well-being” according to the JD-R model	
	JD-R outcomes	Picklist Outcomes: the outcome fits with “outcomes” according to the JD-R model N/A: the outcome does not fit with “outcomes” according to the JD-R model	
**4. Psychometric properties**			
	Validity	Picklist Valid: the instrument is tested and proven valid in the study (eg, content validity, construct validity, criterion validity) [26] N/A: validity is not tested in the study	
	Reliability	Picklist Reliable: the instrument is tested and proven reliable in the study (eg, internal consistency, reliability, measurement error) [26]	
	Responsiveness	Picklist Responsiveness: the instrument is responsive (the ability of the instrument to detect relevant changes in health status when they exist) [26] N/A: responsiveness is not tested in the study	
	Quality reference	Only for reference(s) to the original evaluation study; formatted as author; year	

^a^JD-R: job demands-resources.

^b^N/A: not applicable.

### Risk of Bias

Evaluating evidence quality is not applicable for scoping reviews [22,27]. AB, KvdB, and NA are junior researchers with relatively little experience in conducting scoping reviews on the topic of the well-being of nurses and doctors, but they are supervised by senior researchers with ample experience. The variety of backgrounds, opinions, experiences, and perspectives within the interdisciplinary research team supports self-reflectivity about the subjective values, biases, and inclinations of the researchers. In addition to the medical doctors, other health care professional types are also represented within the research team and have experience with the well-being topic. The first author is a nurse and KvdB has a physiotherapy background. Likewise, LH specializes in the methodology of reviews.

### Data Analysis

This scoping review will determine the size and nature of the evidence base for instruments evaluating well-being at work. The analysis will be descriptive and findings will be assimilated, synthesized, and described. The plan is to summarize validated well-being instruments in tables and figures according to the elements of the JD-R model. Additionally, we will report how often instruments have been extracted, which aspects of well-being are measured, and which types of health care professional are targeted. Synthesizing the data, a distinction will be made between comprehensive instruments and common instruments. The more JD-R domains an instrument encompasses, the more comprehensive (measuring entire well-being) the instrument will be considered to be. Most common instruments are instruments occurring frequently. Results will help readers interpret and choose fitting instruments.

### Ethical Considerations

This study does not require ethical approval since it is a literature review.

## Results

Studies were searched between September 2021 and December 2021. Studies were screened between December 2021 and May 2022. A total of 739 studies were included. Thereafter, data extraction and full text screening were conducted at the same time. The protocol was submitted for publication in October 2022. Before submission, we carried out our search and started screening and extracting data from relevant articles but had no insight into the overall data and had not yet performed data analysis.

## Discussion

This scoping review gives an overview of instruments that have been developed and validated for monitoring the well-being of nurses and doctors at work.

Published research shows a great variety in concepts and measures of well-being at work in terms of target population (professions), settings, and aspects of well-being at work. However, a critical, overarching analysis of these concepts and measures is missing in the scientific literature. There is often a substantial gap and mismatch between employer perceptions of well-being and well-being interventions [8]. It is important to develop and implement suitable interventions adapted to the needs of nurses and doctors and their health or other problems [8,17]. Timely screening of well-being is necessary to gain insight into these needs and problems. Moreover, it is necessary to determine the effectiveness of well-being interventions [8,17].

Our study has some limitations that need to be considered. First, the articles will be divided among 3 researchers because of the amount of data. Consequently, data screening and extraction will be performed once. In order to avoid bias, pilot sets will be screened until consensus on eligibility criteria and JD-R classification is reached. Additionally, an audit trail is used to describe and link thoughts, doubts, and methodological choices.

This study is distinctive in that it does not focus specifically on one instrument but provides an overview of many instruments. By using the JD-R model, we are able to determine which instruments fit in the construct of well-being. By categorizing the instruments according to the elements of the JD-R model, the reader will be supported in selecting instruments appropriate to their context.

## Abbreviations

JD-R: job demands-resources
MeSH: Medical Subject Heading
NFU: Netherlands Federation of University Medical Hospitals
PRISMA-ScR: Preferred Reporting Items for Systematic reviews and Meta-Analyses extension for Scoping Reviews

## Data Availability

All data generated or analyzed during this study will be included in the published article or its supplementary information files.
